# Effects of Cortisol and Dexamethasone on Insulin Signalling Pathways in Skeletal Muscle of the Ovine Fetus during Late Gestation

**DOI:** 10.1371/journal.pone.0052363

**Published:** 2012-12-26

**Authors:** Juanita K. Jellyman, Malgorzata S. Martin-Gronert, Roselle L. Cripps, Dino A. Giussani, Susan E. Ozanne, Qingwu W. Shen, Min Du, Abigail L. Fowden, Alison J. Forhead

**Affiliations:** 1 Department of Physiology, Development and Neuroscience, University of Cambridge, Cambridge, United Kingdom; 2 Metabolic Research Laboratories, Institute of Metabolic Science, University of Cambridge, Cambridge, United Kingdom; 3 Department of Animal Science, University of Wyoming, Laramie, United States of America; Universitat de Barcelona, Spain

## Abstract

Before birth, glucocorticoids retard growth, although the extent to which this is mediated by changes in insulin signalling pathways in the skeletal muscle of the fetus is unknown. The current study determined the effects of endogenous and synthetic glucocorticoid exposure on insulin signalling proteins in skeletal muscle of fetal sheep during late gestation. Experimental manipulation of fetal plasma glucocorticoid concentration was achieved by fetal cortisol infusion and maternal dexamethasone treatment. Cortisol infusion significantly increased muscle protein levels of Akt2 and phosphorylated Akt at Ser473, and decreased protein levels of phosphorylated forms of mTOR at Ser2448 and S6K at Thr389. Muscle GLUT4 protein expression was significantly higher in fetuses whose mothers were treated with dexamethasone compared to those treated with saline. There were no significant effects of glucocorticoid exposure on muscle protein abundance of IR-β, IGF-1R, PKCζ, Akt1, calpastatin or muscle glycogen content. The present study demonstrated that components of the insulin signalling pathway in skeletal muscle of the ovine fetus are influenced differentially by naturally occurring and synthetic glucocorticoids. These findings may provide a mechanism by which elevated concentrations of endogenous glucocorticoids retard fetal growth.

## Introduction

In all species studied to date, there is an increase in the circulating glucocorticoid concentration in the fetus near term [1]. Glucocorticoids such as cortisol promote differentiation of specific cell types in fetal tissues at the expense of cell proliferation and growth. They are thereby responsible for both the normal decrease in fetal growth rate that occurs near term and the prepartum maturation of tissues essential for the successful transition from the intrauterine to the extrauterine environment at birth, including insulin-sensitive tissues such as skeletal muscle [1], [2]. The maturational properties of glucocorticoids are beneficial in clinical practice where synthetic glucocorticoids such as dexamethasone are routinely administered to pregnant women at risk of preterm delivery in order to accelerate fetal maturation and improve survival and health outcomes of the premature neonate [3]. However, exposure of the fetus to antenatal glucocorticoid treatment, and endogenous glucocorticoids elevated in response to suboptimal intrauterine conditions, may impair fetal growth and alter insulin sensitivity and glucose metabolism in the adult animal [4], [5], [6].

The insulin receptor (IR) and insulin-like growth factor type 1 receptor (IGF-1R) are members of the family of receptor tyrosine kinases. When activated by insulin or IGFs, these kinases phosphorylate insulin receptor substrates (IRS) which, in turn, lead to the activation of a number of cascades, including the phosphophatidylinositol 3-kinase (PI3K) signalling pathway. This pathway can induce Akt phosphorylation which induces tissue-specific metabolic effects, such as translocation of the glucose transporter-4 (GLUT4) and consequently stimulation of glucose uptake in skeletal muscle and adipose tissue, activation of the peroxisome proliferator activated receptors involved in fat metabolism and storage, and stimulation of glycogen synthase activity and glycogen deposition in skeletal muscle and liver [7], [8]. Activation of the PI3K pathway also leads to phosphorylation of mammalian target of rapamycin (mTOR) which controls the phosphorylation rate of proteins such as ribosomal protein S6 kinase (S6K) that in turn promotes protein translation [7], [8]. Tissue mTOR expression thereby has an important role in the coordination of nutrient status, protein synthesis and tissue growth [9], [10].

In fetal life, insulin has both glucoregulatory and anabolic actions from at least mid-gestation and developmental changes in the expression of insulin receptors and intracellular signalling proteins have been observed in fetal tissues, including skeletal muscle [11]–[14]. For example, the expression of GLUT4 in skeletal muscle of rats and baboons is lower in fetal compared to adult life and increases with gestational age [13], [14]. However, little is known about the regulation of insulin signalling pathways by glucocorticoids before birth. Therefore, the aim of the present study was to determine the effect of endogenous and synthetic glucocorticoids on the expression of insulin signalling proteins in skeletal muscle of fetal sheep. Glucocorticoid exposure *in utero* was hypothesised to decrease the abundance of proteins associated with growth, such as mTOR and S6K, and increase the proteins associated with glucose transport, such GLUT4. These changes may be responsible for the decline in fetal growth towards term and the preparation for greater dependence on insulin-stimulated glucose disposal after birth, respectively. Experimental manipulations of fetal plasma concentrations of glucocorticoid were achieved by fetal cortisol infusion and maternal dexamethasone treatment. These two experimental models enabled the impact of glucocorticoid exposure on insulin signalling proteins to be assessed.

## Methods

### Animals

Twenty-one Welsh Mountain sheep fetuses of known gestational age were used in this study; all were singleton fetuses (13 male and 8 female). The ewes were housed in individual pens and fed concentrates (200 g day^−1^; 18% protein and 10 MJ kg^−1^; Sheep Nuts #6; H&G Beart, Kings Lynn, UK) with free access to hay, water and a salt-lick block. Overall, the pregnant ewes consumed between 8–11 MJ day-1 of metabolizable energy. Food, but not water, was withheld for 18–24 h before surgery. All surgical and experimental procedures were conducted in accordance with the UK Animals (Scientific Procedures) Act 1986 and were approved by the University of Cambridge animal ethics committee.

### Surgical and Experimental Procedures

#### Fetal cortisol infusion

Under halothane anesthesia (1.5% in O_2_-N_2_O), catheters were implanted into the femoral artery and vein of eleven fetuses at 115–117 days of gestation using surgical methods described previously (term 145±2 days) [15]. Blood samples (2 ml) were taken daily throughout the experimental period from the catheterized fetuses to monitor fetal blood gas status. At least six days after catheterization, the fetuses were infused intravenously with either cortisol (2–3 mg kg^−1^day^−1^ in 3.0 ml 0.9% saline; EF-Cortelan, GlaxoSmithKline, Brentford, Middlesex, UK; n = 5) or the saline vehicle (3.0 ml day^−1^ 0.9% wt/vol; n = 6) for five days beginning at 125–126 days of gestation. The dose of cortisol was chosen to mimic the plasma concentrations normally observed in the immediate prepartum period in fetal sheep [1].

#### Maternal dexamethasone treatment

In a separate cohort of animals, ten un-operated pregnant ewes were injected twice i.m. with either dexamethasone (12 mg dexamethasone sodium phosphate; Merck Sharpe and Dohme Ltd, Hoddesdon, Herts, UK; n = 5) in 2 ml saline or the saline vehicle (0.9% w/v NaCl; n = 5) at 24 hour intervals from 125 days of gestation. Tissues were collected 10 hours after the final maternal injection. The dexamethasone treatment regime resembles that recommended for use in human clinical practice [16].

### Blood and Tissue Collection

All fetuses were delivered by Caesarean section under general anaesthesia of the ewe (20 mg kg^−1^ sodium pentobarbitone i.v.). At delivery, a 10 ml blood sample was taken by venepuncture of the umbilical artery, and a number of tissues were collected from the fetus after the administration of a lethal dose of barbiturate (200 mg kg^−1^ sodium pentobarbitone). Skeletal muscle tissue samples taken from the hind limb (biceps femoris) were immediately frozen in liquid nitrogen and stored at −80°C until analysis. All blood samples obtained at tissue collection were immediately placed into EDTA-containing tubes and centrifuged for 5 min at 1000 x g and 4°C. The plasma aliquots were stored at –20°C until analysis.

### Biochemical Analyses

Plasma cortisol concentration was measured in the umbilical arterial sample by radioimmunoassay validated for use with ovine plasma [17]. The lower limit of detection was 1.0–1.5 ng.ml^−1^, and the inter-assay coefficient of variation was 12%.

Muscle protein levels were quantified by Western blotting using methods described previously [18], [19]. The antibodies used in this study were to IR-β subunit, IGF-1R, PKCζ, Akt1, Akt2, p-Akt Ser473, p-mTOR Ser2448, p-S6K Thr389, calpastatin and GLUT4 (Table 1), and have been used previously in this laboratory to quantify protein levels in the skeletal muscle of fetal sheep [20]. Horseradish peroxidase-conjugated secondary antibodies to the host species were used as appropriate and antibody binding was detected using an enhanced chemiluminescence kit (all products from GE Healthcare Life Sciences, Little Chalfont, Bucks, UK). Protein expression was quantified using Image J software (National Institutes of Health, Bethesda, MD; http://rsb.info.nih.gov/ij/).

**Table 1:** Details of the antibodies used in Western blotting.

Protein	Antibody	Dilution	Source	Catalogue Number
IR-β	Rabbit polyclonal	1∶200	Santa Cruz Biotechnologies, Santa Cruz, CA, USA	sc711
IGF-1R	Rabbit polyclonal	1∶200	As above.	sc713
PKCζ	Rabbit polyclonal	1∶500	As above.	sc216
p-Akt Ser473	Rabbit monoclonal	1∶1000	Cell Signaling Technology, Inc, Beverly, MA, USA	4058
Akt1	Mouse monoclonal	1∶1000	As above.	2967
Akt2	Rabbit monoclonal	1∶1000	As above.	3063
p-mTOR Ser2448	Rabbit polyclonal	1∶1000	As above.	2971
p-S6K Thr389	Rabbit polyclonal	1∶1000	As above.	9205
Calpastatin	Chicken polyclonal	1∶1000	Abcam, Cambridge, UK	ab16423
GLUT4	Rabbit polyclonal	1∶4000	As above.	ab654

The glycogen content of skeletal muscle was determined by a biochemical assay as described previously [21]. The inter-assay coefficient of variation was 9%.

### Statistical Analyses

Values are presented as mean values ± SEM. Data were analysed using Student’s t-test. Ratios of muscle protein expression were arcsine transformed prior to statistical analysis, and are presented as a fold change from the mean values observed in the respective control group. For all comparisons, statistical significance was accepted when *P<*0.05.

## Results

### Fetal Cortisol Infusion

**Figure 1:**
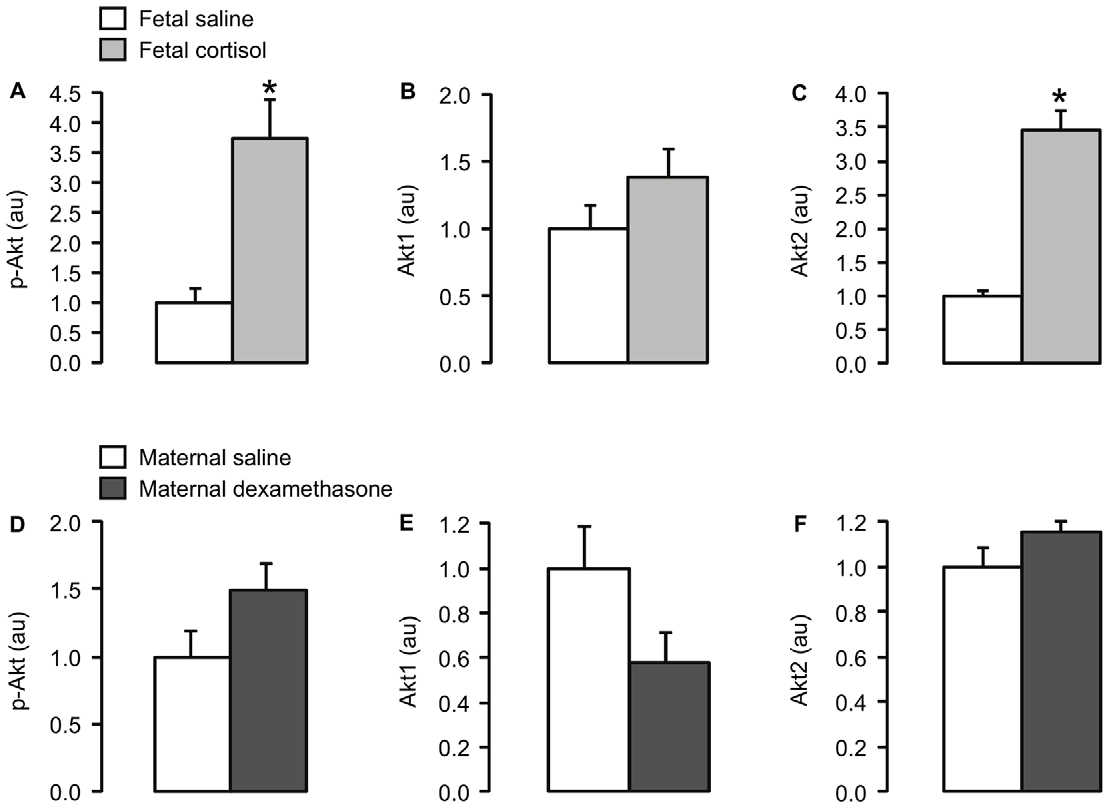
Mean (± SEM) protein levels of (A, D) p-Akt, (B, E) Akt1 and (C, F) Akt2 in skeletal muscle of fetuses infused i.v. with either saline or cortisol for 5 days, and fetuses whose mothers were injected i.m. with either saline or dexamethasone. *, significantly different from saline control group, p<0.05.

**Figure 2:**
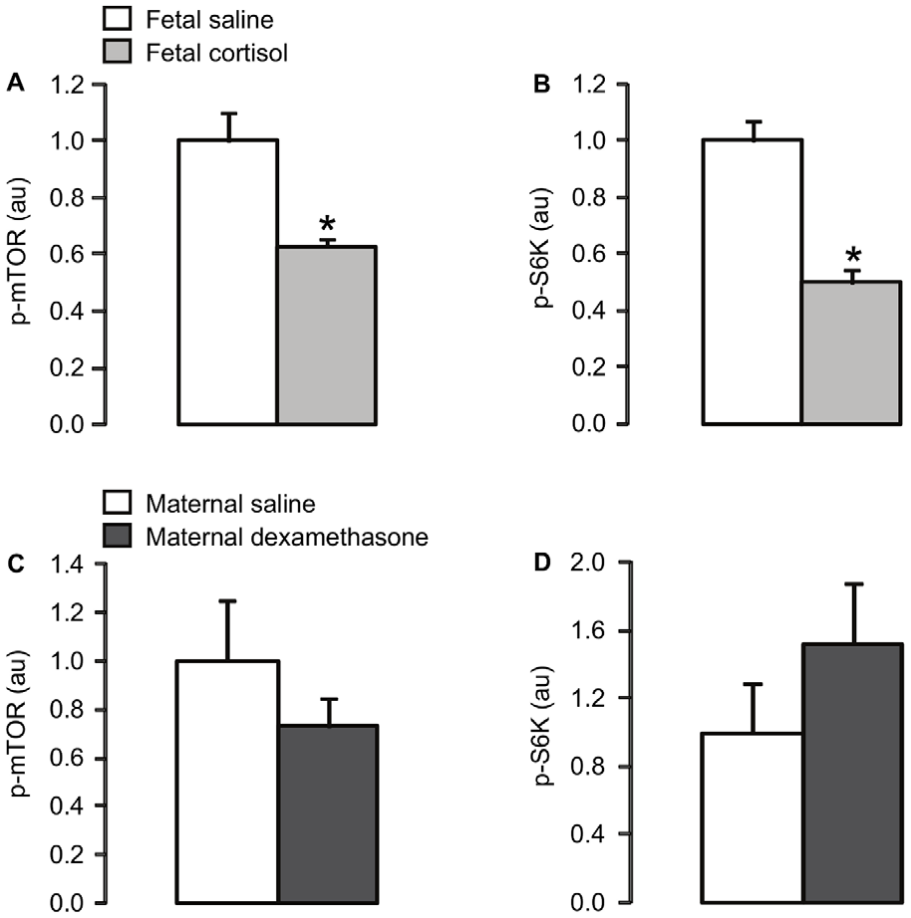
Mean (± SEM) protein levels of (A, C) p-mTOR and (B, d) p-S6K in skeletal muscle of fetuses infused i.v. with either saline or cortisol for 5 days, and fetuses whose mothers were injected i.m. with either saline or dexamethasone. *, significantly different from saline control group, p<0.05.

**Figure 3:**
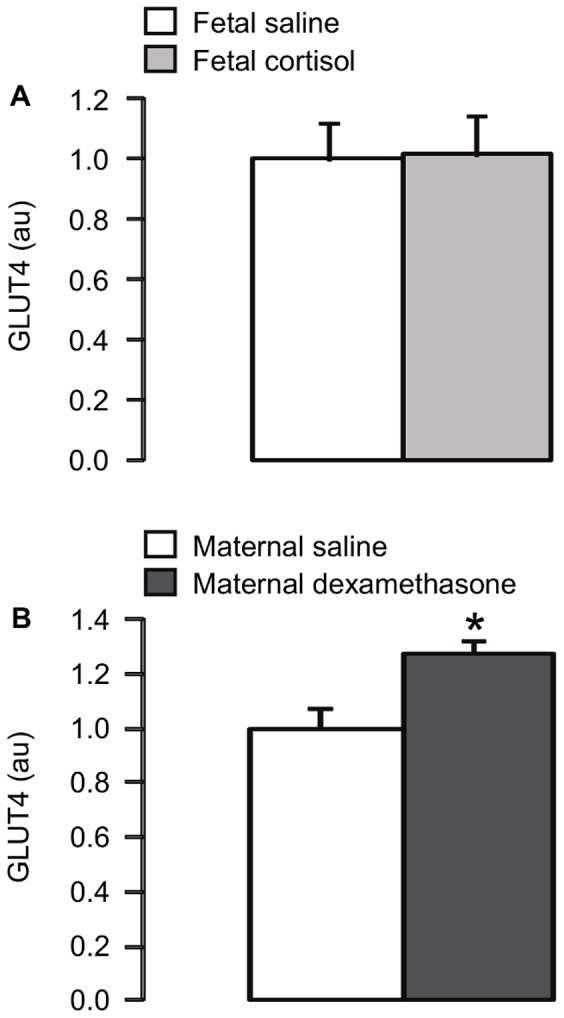
Mean (± SEM) GLUT4 protein level in skeletal muscle of (a) fetuses infused i.v. with either saline or cortisol for 5 days, and (b) fetuses whose mothers were injected i.m. with either saline or dexamethasone. *, significantly different from saline control group, p<0.05.

Plasma cortisol concentration was significantly higher in the cortisol-infused fetuses than in the saline-infused fetuses (p<0.05; Table 2). Fetal cortisol infusion for five days led to significant increases in the muscle protein level of p-Akt Ser473 and the expression of Akt2 (p<0.05), but not Akt1 (Figure 1A, B and C), and decreases in the protein expression of p-mTOR Ser2448 and p-S6K Thr389 (p<0.05; Figure 2A and B). There were no significant differences in muscle protein abundance of IR-β, IGF-1R, PKCζ, calpastatin (Table 2) or GLUT4 (Figure 3B), or muscle glycogen content (Table 2), between the saline and cortisol-treated fetuses.

**Table 2:** Mean (± SEM) plasma concentrations of cortisol, muscle protein levels of IR-β, IGF-1R, PKCζ and calpastatin, and muscle glycogen content in fetuses infused i.v. with either saline or cortisol for 5 days, and in fetuses whose mothers were injected i.m. with either saline or dexamethasone.

	Fetal administration		Maternal administration	
	Saline	Cortisol	Saline	Dexamethasone
Gestational age (days)	130±0	131±0.2	127±0	127±0
Plasma cortisol (ng.ml^−1^)	14.1±2.0	91.2±20.9*	16.1±2.8	8.6±0.7*
Muscle IR-β (au)	1.00±0.20	1.11±0.10	1.00±0.06	0.88±0.06
Muscle IGF-1R (au)	1.00±0.21	1.06±0.13	1.00±0.16	0.84±0.13
Muscle PKCζ (au)	1.00±0.09	0.86±0.06	1.00±0.03	1.02±0.04
Muscle calpastatin (au)	1.00±0.09	0.83±0.10	1.00±0.04	1.07±0.13
Muscle glycogen (mg.g tissue^−1^)	46.27±3.72	48.38±2.21	48.70±1.95	50.51±3.14

* , significantly different from the respective saline control group, Student’s t-test, p<0.05. IR-β, insulin receptor β-subunit; IGF-1R, insulin-like growth factor-1 receptor; PKCζ, protein kinase C ζ; au, arbitrary units.

### Maternal Dexamethasone Treatment

At delivery, plasma cortisol concentration was significantly lower in fetuses whose mothers were injected with dexamethasone compared to those whose mothers were treated with saline (p<0.05; Table 2). Exposure of fetuses to dexamethasone caused a significant increase in muscle GLUT4 protein content (p<0.05; Figure 3B). However, fetal muscle protein levels of IR-β, IGF-1R, PKCζ, calpastatin (Table 2), Akt 1, Akt2 and p-Akt Ser473 (Figure 1D, E and F), p-mTOR Ser2448 and p-S6K Thr389 (Figure 2C and D), and muscle glycogen content (Table 2) were not significantly different between the saline and dexamethasone-treated animals.

## Discussion

The results demonstrate that components of the insulin signalling pathways relating to both growth and glucoregulation in skeletal muscle of the ovine fetus are sensitive to glucocorticoid exposure *in utero*. In agreement with the hypothesis of the study, fetal infusion of cortisol led to downregulation of the phosphorylated levels of the anabolic proteins, mTOR and S6K, while maternal dexamethasone treatment increased GLUT4 expression in fetal skeletal muscle.

These findings contribute to the understanding of the regulation of growth by glucocorticoids *in utero*. In fetal sheep, the decline in growth rate seen near term is known to be glucocorticoid-dependent, since it can be prevented by removal of the fetal adrenal gland and induced prematurely by five days of cortisol infusion [2]. Reductions in the phosphorylated levels of mTOR and S6K induced by cortisol in skeletal muscle of the ovine fetus may be one mechanism by which glucocorticoids retard muscle protein synthesis and overall fetal growth. Indeed, glucocorticoids have been shown previously to decrease S6K phosphorylation and protein synthesis in cultured L6 myoblasts and in rat skeletal muscle *in vivo* [22], [23].

In the present study, reductions in muscle p-mTOR and p-S6K in the cortisol-infused fetuses were associated with increased Akt2 and p-Akt protein content. This finding was unexpected given that the PI3K/Akt pathway is a key activator of mTOR phosphorylation in adult tissues [7], [8]. It is possible that the relative levels of phosphorylation of Akt at Ser473 and Thr308 may change in response to glucocorticoid treatment with consequences for the specificity of downstream targets. Consistent with this possibility, a recent study in the Jeg-3 human choriocarcinoma cell line has shown that an increase in Akt phosphorylation at Ser473, induced by endoplasmic reticulum stress, decreases phosphorylation of mTOR at Ser2448 while increasing phosphorylation of other protein targets [24]. In addition, cortisol may influence p-mTOR and p-S6K expression independently of the PI3K/Akt pathway and/or downstream of these proteins in fetal skeletal muscle. For example, glucocorticoids have been shown to suppress mTOR levels, at least in part, by activation of the translation repressor protein REDD1 in cultured L6 myoblasts and in rat skeletal muscle *in vivo* [25]. There is also evidence to suggest that mTOR has negative feedback effects on Akt phosphorylation. Inhibition of mTOR using rapamycin or its derivatives upregulates Ser473 phosphorylation of Akt in cancer cell lines and human tumours [26], [27]. Of the three isoforms of Akt, Akt2 is highly expressed in insulin-sensitive tissues, such as skeletal muscle, and previously, increased Akt2 expression has been associated with increased myoblast differentiation *in vitro* [28], [29]. In the present study, however, the tissues were not examined histologically and the effect of cortisol exposure on the structural development of the biceps femoris *in utero* remains unclear.

Muscle GLUT4 protein expression in the ovine fetus was increased by maternal dexamethasone treatment. This observation confirms that previously reported in fetuses of pregnant ewes treated with either single or repeated doses of dexamethasone at an earlier gestational age (106–107 days) [30]. Dexamethasone has also been shown to increase GLUT4 protein levels in cultured L6 myoblasts and in rat skeletal muscle *in vivo* [31], [32]. Upregulation of muscle GLUT4 in the sheep fetus by maternal dexamethasone treatment occurred without any effect on other components of the insulin signalling pathway, such as p-Akt and PKCζ. Muscle GLUT4 content in the fetus may be influenced by insulin-independent as well as insulin-dependent mechanisms. For example, in fetal sheep, both experimental conditions of hyperglycaemia-euinsulinaemia and hyperinsulinaemia-euglycaemia increase GLUT4 protein expression in skeletal muscle in a time-dependent manner [33]. Despite the increase in muscle GLUT4 protein level observed in the sheep fetuses exposed to dexamethasone, there were no changes in muscle glycogen although glycogen deposition also depends on intracellular glucose availability, as well as the activities of glycogen synthase and phosphorylase enzymes which were not determined in the present study. Furthermore, the extent to which GLUT4 translocation to the muscle plasma membrane is affected by glucocorticoids *in utero* remains unknown.

Cortisol and dexamethasone exerted different effects on muscle insulin signalling proteins in the fetuses of the two experimental models of intrauterine glucocorticoid exposure. There are several possible explanations for these observations. First, the experimental models differ in the duration and level of glucocorticoid exposure and timing of tissue collection. In the dexamethasone-treated animals, dexamethasone concentrations in the fetal circulation are significantly elevated 1–2 h post-injection and, although still detectable, fall thereafter at tissue and blood collection at 10 hours after the second maternal injection [34], whereas cortisol-treated fetuses were exposed to elevated and stable levels of glucocorticoid for five days prior to tissue collection. In both models, there may have been dynamic changes in insulin signalling protein expression over the time course of each treatment.

Second, natural and synthetic glucocorticoids differ in their properties of action. The synthetic glucocorticoids have longer half-lives and are more potent than the endogenous glucocorticoids, and, unlike the endogenous glucocorticoids that act on both glucocorticoid and mineralocorticoid receptors, synthetic glucocorticoids only bind and activate glucocorticoid receptors. The relative effects of activation of mineralocorticoid receptors by cortisol on insulin signalling pathways in fetal tissues, however, are unknown. The fall in plasma cortisol seen in the dexamethasone-exposed fetuses is in accordance with the negative feedback effects of synthetic glucocorticoids on endogenous glucocorticoid production and has been reported previously in the fetuses of pregnant ewes treated with dexamethasone [21].

Third, some of the effects of glucocorticoid exposure on insulin signalling protein expression in skeletal muscle *in utero* may be due to concomitant changes in other circulating and/or local hormones and growth factors. In both of these fetal sheep models, increases in plasma triiodothyronine and leptin have been demonstrated in response to fetal cortisol infusion and maternal dexamethasone treatment [35], [36], [37]. Although components of the insulin signalling pathway in fetal skeletal muscle do not appear to be influenced by leptin treatment *in utero* [20], the effects of triiodothyronine are unknown. In addition, previous studies have shown that maternal dexamethasone treatment in pregnant ewes causes increases in circulating glucose and insulin in the fetus [21], while no changes in plasma glucose and insulin are observed after five days of fetal cortisol infusion [2]. The insulin response to dexamethasone may negate some of the direct actions of the glucocorticoid on the insulin signalling proteins as insulin promotes expression of anabolic proteins, such as S6K, in skeletal muscle of the ovine fetus [38]. Furthermore, in fetal sheep, both glucose and insulin are able to upregulate muscle GLUT4 protein expression [33] and, therefore, the dexamethasone-induced increments in plasma glucose and insulin are likely to contribute to the increase in muscle GLUT4 seen in the present study.

Suppression of mTOR and S6K in skeletal muscle of cortisol-treated sheep fetuses may also be due, at least in part, to local changes in IGF synthesis. Previous studies have shown that cortisol infusion for five days in fetal sheep downregulates both IGFI and IGFII mRNA abundance in skeletal muscle [39], [40] which may reduce the anabolic actions of these paracrine agents in the absence of any change in muscle IGF-1R expression. Muscle IGF expression has not been measured in the experimental model of dexamethasone exposure used in the present study, although in other studies, synthetic glucocorticoid treatment in the pregnant ewe has been shown to reduce circulating IGFI and IGF binding proteins in the fetus only after repeated, but not single, doses [41]. Furthermore, in fetal sheep and human infants exposed to synthetic glucocorticoids *in utero*, multiple doses lead to impaired body size at birth while a single dose often has little effect on growth [42], [43].

In summary, the current study demonstrated that endogenous and synthetic glucocorticoids alter components of the insulin signalling pathways in skeletal muscle of the ovine fetus in different ways, with potentially different consequences for the regulation of muscle growth in the fetus and insulin sensitivity in postnatal life. An infusion of cortisol to the fetus for five days reduced phosphorylated levels of mTOR and S6K which, in turn, are likely to contribute to the mechanisms of glucocorticoid-induced growth retardation. In contrast, a single course of maternal dexamethasone treatment had little effect on the insulin signalling molecules measured in fetal skeletal muscle, apart from an increase in GLUT4 expression which may be a homeostatic response to the fetal hyperglycaemia seen previously after dexamethasone exposure [21]. These findings suggest that synthetic glucocorticoid treatment during pregnancy may have minimal consequence for insulin signalling and growth of skeletal muscle in the fetus, at least in the short term.
